# Protracted osilodrostat-induced pan-adrenal steroidogenic suppression and adrenal size reduction in Cushing disease

**DOI:** 10.1210/jcemcr/luag178

**Published:** 2026-07-23

**Authors:** Yael Sofer, Rivka Kessner, Sher Matsri, Yona Greenman, Karen M Tordjman

**Affiliations:** Institute of Endocrinology, Metabolism and Hypertension, Tel Aviv Sourasky Medical Center, Tel Aviv 640239, Israel; Gray Faculty of Medical and Health Sciences, Tel Aviv University, Tel Aviv 6997801, Israel; Gray Faculty of Medical and Health Sciences, Tel Aviv University, Tel Aviv 6997801, Israel; Department of Radiology, Tel Aviv Sourasky Medical Center, Tel Aviv 640239, Israel; Institute of Endocrinology, Metabolism and Hypertension, Tel Aviv Sourasky Medical Center, Tel Aviv 640239, Israel; Institute of Endocrinology, Metabolism and Hypertension, Tel Aviv Sourasky Medical Center, Tel Aviv 640239, Israel; Gray Faculty of Medical and Health Sciences, Tel Aviv University, Tel Aviv 6997801, Israel; Institute of Endocrinology, Metabolism and Hypertension, Tel Aviv Sourasky Medical Center, Tel Aviv 640239, Israel; Gray Faculty of Medical and Health Sciences, Tel Aviv University, Tel Aviv 6997801, Israel

**Keywords:** Cushing disease, osilodrostat, prolonged adrenal insufficiency, pan-adrenal steroidogenic suppression, adrenal gland shrinkage

## Abstract

Osilodrostat, a potent 11β-hydroxylase inhibitor, is used for Cushing syndrome. Transient adrenal insufficiency during dose titration is common and usually reversible, whereas prolonged adrenal insufficiency after treatment discontinuation appears uncommon. A man with treatment-resistant Cushing disease was treated with osilodrostat after prior transsphenoidal surgeries and stereotactic radiosurgery. He initially demonstrated the expected biochemical profile of 11β-hydroxylase inhibition, with elevated adrenocorticotropic hormone (ACTH) and 11-deoxycortisol concentrations. After 18 months of therapy, he developed adrenal insufficiency with morning cortisol 1.27 μg/dL (SI: 35 nmol/L) [reference 5-22.6 μg/dL; 145-619 nmol/L], ACTH 989 pg/mL (SI: 217.6 pmol/L) [reference 4-46 pg/mL; 1.0-10.1 pmol/L], suppressed aldosterone 1.2 ng/dL (SI: 33.2 pmol/L) [reference 2-35 ng/dL; 55.4-970 pmol/L], markedly elevated direct renin 288.5 mIU/mL [reference 4.4-46.1 mIU/mL], and suppression of adrenal androgen production. Osilodrostat was discontinued and glucocorticoid replacement initiated, with later introduction of mineralocorticoid replacement. At 2.5 years, glucocorticoid and androgen suppression persist, together with prolonged mineralocorticoid dysfunction and adrenal size reduction. Review of 10 previous reports suggests prolonged adrenal insufficiency is uncommon and mineralocorticoid deficiency rarely documented, while persistent androgen suppression has not previously been reported. This case highlights prolonged pan-adrenal steroidogenic suppression with adrenal shrinkage and suggests recovery of adrenal function may take years.

## Introduction

Cushing disease (CD) is associated with multiple comorbidities and reduced life expectancy, that may persist even after biochemical remission. It is caused by an adrenocorticotropic hormone (ACTH)-secreting pituitary tumor, and first-line treatment is transsphenoidal surgery (TSS) [1]. Surgical remission rates range from 60% to 80%, depending on tumor size, invasiveness, and surgical expertise. The presence of an ACTH-positive adenoma on pathology remains the most reliable predictor of remission [2]. Even after remission, recurrence occurs in about 25% of patients within 10 years [3].

Minute tumors may escape detection on imaging, leading to failed surgery despite pituitary origin confirmed by inferior petrosal sinus sampling [1]. Reoperation can be successful, although remission rates are roughly half those of initial surgery. Patients with persistent or recurrent disease require additional therapy.

Stereotactic pituitary irradiation can induce remission in up to 80% of such patients [3], but concerns regarding hypopituitarism and optic nerve injury limit its use. Medical therapy suitable for long-term administration has therefore become increasingly important. Available agents target adrenal steroidogenesis, glucocorticoid receptors, or the pituitary tumor via dopamine or somatostatin receptors [4, 5].

Osilodrostat, a potent inhibitor of 11β-hydroxylase and aldosterone synthase, is a recent addition [6]. Its strong cortisol-lowering effect may induce early hypocortisolemia during titration in about one-third of patients, usually resolving with dose adjustment or a block-and-replace approach [6].

We describe an unusual case of resistant CD more than 10 years after 2 TSS procedures and stereotactic radiosurgery, in which osilodrostat induced pan-adrenal steroidogenic suppression that is still ongoing two-and-a-half years after drug discontinuation. We also review the available literature on this rare occurrence to alert clinicians to its possibility and to outline its main features.

## Case presentation

Admitted in April 2012 with acute visual disturbance, this 56-year-old man was diagnosed with central serous chorioretinopathy [7]. He had a 2-year history of 10-kg weight gain, hypertension, plethora, easy bruising, irritability, and agitation. A diagnosis of Cushing syndrome was contemplated.

## Diagnostic assessment

Cushing syndrome was confirmed as ACTH-dependent: urinary free cortisol (UFC) 752-676 µg/24 hours (SI: 2075-1865 nmol/24 hours), [reference ≤75 µg/24 hours; ≤207 nmol/24 hours]. Serum cortisol after 1 mg dexamethasone 20.6 µg/dL (SI: 568 nmol/L); [reference <1.8 µg/dL; <50 nmol/L]; and ACTH 176 pg/mL (SI: 38.7 pmol/L). He also had central hypogonadism and osteoporosis (lumbar T score −3.0).

Pituitary magnetic resonance imaging showed a 6-mm right-sided lesion. Inferior petrosal sinus sampling confirmed a pituitary source with right lateralization.

## Treatment

In July 2012 he underwent TSS, yielding minute ACTH-positive adenoma fragments. Postoperative morning cortisol was 3.8 µg/dL (SI: 105 nmol/L), and hydrocortisone replacement was initiated. One month later, his UFC was 27.5 µg/24 hours (SI: 76 nmol/24 hours). Five months postoperatively, adrenal function recovered and replacement was stopped.

By early 2013, biochemical relapse was evident despite improvement in central serous chorioretinopathy. Ketoconazole, titrated to 800 mg daily, failed to control hypercortisolism. A second TSS in September 2013 yielded no relevant tissue. In December 2013, he received 17 Gy stereotactic radiosurgery, and cabergoline 0.5 mg twice weekly was added to ketoconazole.

Over the following years, radiation combined with dual medical therapy produced partial biochemical improvement. Hypertension, dyslipidemia, and osteoporosis remained adequately controlled with treatment, he maintained a normal and stable weight, and overt cushingoid features were no longer clinically apparent. However, psychiatric instability, depression, and anxiety, persisted despite pharmacologic treatment. Secondary hypogonadism was present, but the patient declined testosterone replacement.

At the end of 2021, biochemical disease activity worsened. Magnetic resonance imaging suggested tumor recurrence, but the patient declined surgery. Osilodrostat monotherapy was planned. While awaiting availability, ketoconazole dose was increased from 600 to 800 mg a day, and cabergoline doubled to 2 mg a week, but severe impulse control disorder developed, leading to its discontinuation.

Osilodrostat was initiated in March 2022 at 1 mg twice daily and titrated over 5 months to 10 mg twice daily due to persistent hypercortisolism. By August 2022, serum morning cortisol was 6.3-18.1 µg/dL (SI: 175-500 nmol/L), midnight salivary cortisol was at the upper limit of normal, ACTH increased to 270-346 pg/mL (SI: 59.4-76.1 pmol/L), 11-deoxycortisol rose to 41.8 µg/dL (SI: 1206 nmol/L) [reference 0-20 µg/dL; 0-577 nmol/L], and aldosterone was suppressed <0.97 ng/dL (SI: < 26.9 pmol/L). Renin was not measured. Blood pressure was normal, and the small dose of valsartan (20 mg daily) monotherapy he was still receiving was discontinued and never reinitiated.

In March 2023, UFC normalized at 46 µg/24 hours (SI: 127 nmol/24 hours), 11-deoxycortisol increased further to 46.4 µg/dL (SI: 1336 nmol/L), ACTH rose to 585 pg/mL (SI: 129 pmol/L), and neutropenia prompted dose reduction to 5 mg twice daily. At that time, he had lost 6 kg and complained of weakness, although morning serum cortisol remained within the low-normal range (6.3-8.3 µg/dL [SI: 174-229 nmol/L]). ACTH had risen further (638 pg/mL; SI: 140.5 pmol/L) and aldosterone remained suppressed, yet appetite was preserved, diarrhea and hyperpigmentation were absent, and normal electrolytes, glucose, and blood pressure all argued against clinical adrenal insufficiency.

## Outcome and follow-up

In October 2023, while receiving 10 mg of osilodrostat daily, he was admitted with biochemical and clinical adrenal insufficiency. He had lost an additional 4 kg, had anorexia, and presented with hyponatremia (130 mmol/L), while potassium remained within the normal range at 4.39 mmol/L. Morning cortisol was 1.3 μg/dL (SI: 35 nmol/L) and ACTH 989 pg/mL (SI: 217.6 pmol/L). Aldosterone was suppressed at 1.2 ng/dL (SI: 33.2 pmol/L) with markedly elevated direct renin of 288.5 mIU/mL. Blood pressure and glucose remained preserved.

He responded to intravenous hydrocortisone. Osilodrostat was discontinued, and glucocorticoid replacement initiated (hydrocortisone 30 mg daily, later prednisone 7.5 mg, subsequently reduced to 5 mg). Despite apparent recovery of aldosterone to low-normal concentrations, persistently elevated renin and ongoing symptoms of weakness and mild hyponatremia suggested continued mineralocorticoid insufficiency, and fludrocortisone 50 mcg daily was introduced, resulting in normalization of sodium and renin concentrations with symptomatic improvement.

Currently, two-and-a-half years after discontinuation of osilodrostat, he remains clinically stable on glucocorticoid and mineralocorticoid replacement therapy without recurrence of hypertension or overt hypercortisolism. Nevertheless, adrenal suppression persists, with continued hypocortisolism despite marked ACTH elevation, low adrenal androgens, low aldosterone concentrations, and suppressed glucocorticoid precursors (Table 1). Comparison of abdominal computerized tomography (CT) scans obtained at diagnosis and at the time adrenal insufficiency developed demonstrated bilateral adrenal size reduction (Fig. 1).

**Figure 1:**
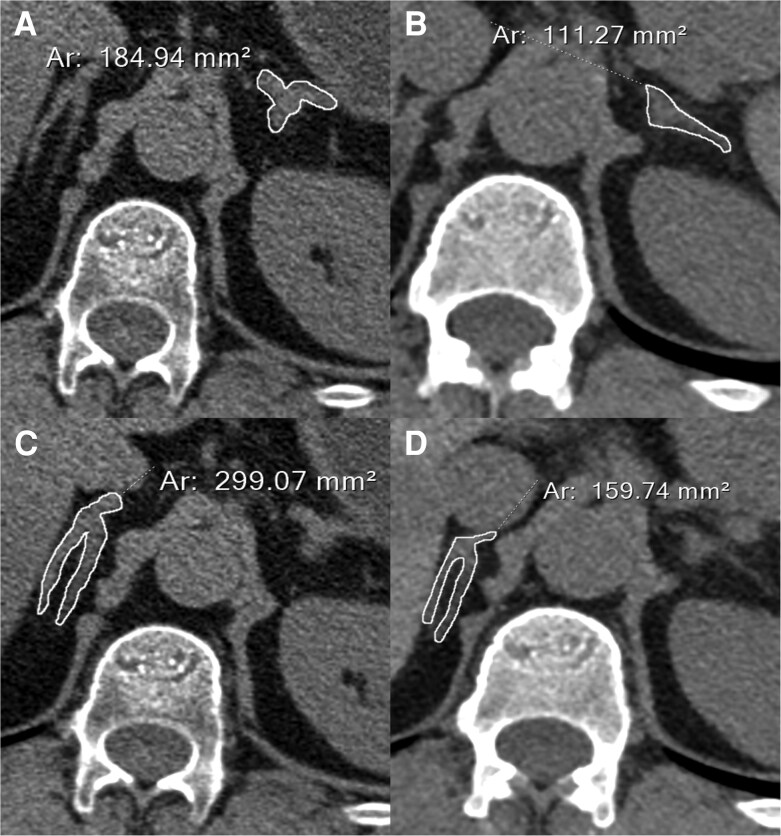
Bilateral adrenal gland size reduction on longitudinal CT imaging. Non-contrast axial CT images showing the left adrenal gland in 2012 (A) and 2023 (B), and the right adrenal gland in 2012 (C) and 2023 (D). On a comparable slice, adrenal area decreased from 184.9 to 111.3 mm^2^ on the left, and from 299.1 to 159.7 mm^2^ on the right. Imaging spans a period before and after exposure to osilodrostat.

**Table 1:** Biochemical findings after initiation of osilodrostat in March 2022

Variable	May 2022	September 2022	March 2023	October 2023	January 2024	October 2024	August 2025	March 2026
Treatment	Osilo 4 mg/d	Osilo 20 mg/d	Osilo 15 mg/d	Osilo 10 mg/d	HC 30 mg/d	HC 30 mg/d	Prednisone 7.5 mg/d	Prednisone 5 mg/d + FC 50 mcg/d
UFC	ND	121.6 µg/24 hours *(336 nmol/24 hours)*	46 µg/24 hours *(127 nmol/24 hours)*	16.8 µg/24 hours *(46 nmol/24 hours)*	ND	ND	ND	ND
Cortisol 08:00	29 µg/dL *(SI: 800 nmol/L)*	14.3 µg/dL *(SI: 394 nmol/L)*	11.9 µg/dL *(SI: 328 nmol/L)*	1.27 µg/dL *(SI: 35 nmol/L)*	2.6 µg/dL *(SI: 72 nmol/L)*	3.1 µg/dL *(SI: 86 nmol/L)*	5.8 µg/dL *(SI: 160 nmol/L)*	5.07 µg/dL *(SI: 140 nmol/L)*
11-deoxycortisol	26.4 µg/dL *(SI: 763 nmol/L)*	41.8 µg/dL *(SI: 1209 nmol/L)*	46.4 µg/dL *(SI: 1342 nmol/L)*	ND	ND	3.3 µg/dL *(SI: 95 nmol/L)*	1.9 µg/dL *(SI: 55 nmol/L)*	4.5 µg/dL *(SI: 130 nmol/L)*
ACTH	ND	270 pg/mL *(SI: 59.4 pmol/L)*	585 pg/mL *(SI: 128.7 pmol/L)*	989 pg/mL *(SI: 217.6 pmol/L)*	231 pg/mL *(SI: 50.8 pmol/L)*	190 pg/mL *(SI: 41.8 pmol/L)*	390 pg/mL *(SI: 85.8 pmol/L)*	429 pg/mL *(SI: 94.4 pmol/L)*
Aldosterone	ND	<0.97 ng/dL *(<27 pmol/L)*	1.0 ng/dL *(27.7 pmol/L)*	1.2 ng/dL *(33.2 pmol/L)*	ND	5.3 ng/dL *(147 pmol/L)*	9.2 ng/dL *(255 pmol/L)*	1.9 ng/dL *(SI: 53 pmol/L)*
Direct renin	ND	ND	ND	288.5 mIU/L	ND	79.7 mIU/L	136.4 mIU/L	45.1 mIU/L
DHEAS *(ref 33-567 µg/dL; SI: 0.9-15.4 µmol/L)*	ND	72 µg/dL *(SI: 1.94 µmol/L)*	29.5 µg/dL *(SI: 0.80 µmol/L)*	ND	<15 µg/dL *(SI:<0.41 µmol/L)*	<15 µg/dL *SI: (<0.41 µmol/L)*	<15 µg/dL *(SI:<0.41 µmol/L)*	18.4 µg/dL *(SI: 0.50 µmol/L)*
Androstenedione *(ref 0.5-3.5 ng/mL; SI: 1.7-12.2 nmol/L)*	ND	6.82 ng/mL *(SI: 23.8 nmol/L)*	7.93 ng/mL *(SI: 27.7 nmol/L)*	ND	ND	ND	0.48 ng/mL *(1.68 nmol/L)*	<0.3 ng/mL *(<1.05 nmol/L)*
Sodium	ND	ND	ND	130 mmol/L	131 mmol/L	135 mmol/L	133 mmol/L	140 mmol/L
WBC *(ref 4.5-11.0 ×10^3^/µL)*	5.1 ×10^3^/µL	3.7 ×10^3^/µL	2.9 ×10^3^/µL	3.0 ×10^3^/µL	4.2 ×10^3^/µL	4.7 ×10^3^/µL	ND	4.2 ×10^3^/µL
Neutrophils *(ref 1.8-8.0 ×10^3^/µL)*	2.9 ×10^3^/µL	2.28 ×10^3^/µL	1.85 ×10^3^/µL	1.7 ×10^3^/µL	2.54 ×10^3^/µL	3.01 ×10^3^/µL	ND	2.46 ×10^3^/µL

Abbreviations: ACTH, adrenocorticotropic hormone; DHEAS, dehydroepiandrosterone sulfate; FC, fludrocortisone; HC, hydrocortisone; ND, not done; Osilo, osilodrostat; UFC, urinary free cortisol.

## Discussion

Osilodrostat is the most potent 11β-hydroxylase inhibitor currently used for treatment of Cushing syndrome. Early adrenal insufficiency occurs in approximately one-third of treated patients and is usually managed with dose reduction, temporary interruption, or a block-and-replace regimen [6, 8]. Osilodrostat inhibits aldosterone synthase (CYP11B2) in vitro as strongly as CYP11B1, and clinical studies have demonstrated marked reductions in aldosterone concentrations during treatment [9, 10]. Nevertheless, clinically significant mineralocorticoid deficiency appears uncommon in routine practice, possibly because accumulation of 11-deoxycorticosterone, a steroid with mineralocorticoid activity, partially compensates for aldosterone suppression.

Prolonged adrenal insufficiency persisting after discontinuation has been reported in 10 patients, excluding the present case [11-17] (Table 2). Adrenal insufficiency developed between less than 4 and 15 months after treatment initiation and persisted from 1.5 to more than 36 months. Most reports were published before recovery of adrenal function or recurrence of hypercortisolism could be assessed. Mineralocorticoid deficiency was absent, not mentioned, or inferred indirectly in only 2 subjects from empirical fludrocortisone treatment rather than documented biochemically [11, 12]. Other adrenal steroid pathways were generally not evaluated in detail. In one patient with CD, dehydroepiandrosterone sulfate (DHEAS) was suppressed while 11-deoxycortisol concentrations were normal [17]. Most previously reported cases lacked detailed longitudinal evaluation of glucocorticoid precursors, adrenal androgens, and mineralocorticoid pathway function, limiting mechanistic interpretation of the prolonged adrenal insufficiency phenotype.

**Table 2:** Characteristics of reported cases of osilodrostat-induced prolonged adrenal insufficiency in patients with Cushing syndrome

First author (Ref.)	Year	Age (y)	Sex	CS type	Treatment duration (months)	Maintenance dose (mg/d)	Mineralocorticoid involvement	Duration of AI (months)	Cortisol status at time of publication
Poirier-patient 1 [11]	2023	51	F	CD	6	2	No	1.5	Rec
Poirier-patient 2 [11]	2023	31	F	CD	15	4	No	12.5	Rec.
Poirier-patient 3 [11]	2023	41	M	ACTH-dependent (cyclic)	13	10	Possible (FC despite normal aldosterone and renin)	9.8	AI
Ferriere-patient 1 [12]	2024	59	M	Ectopic ACTH	6	3	No	12	Partial
Ferriere-patient 2 [12]	2024	51	F	CD	15	1	Possible (FC, normal DOC)	15	AI
Tejani [13]	2024	41	F	CD	11	6	No	23	Norm
Veloski [14]	2025	38	F	ACTH-independent macronodular hyperplasia	<4	20	No	>36	AI
Kaniuka-Jakubowska [15]	2026	74	F	Ectopic ACTH	15	3	Not reported	7.8	AI
Benderradji [16]	2025	81	F	Ectopic ACTH	5	20	Not reported	10	AI
Dzialach [17]	2025	43	M	CD	67.5	0.3	No	5	Norm
Current report	2026	70	M	CD	18	10	Yes	30	AI
All cases (n = 11) median (IQR) or (n/N)		51 (41-70)	F/M 7/4	CD/Non-CD 6/5	13 (6-15)	4 (2-10)	Yes/No 3/6	12 (7.8-23)	

Abbreviations: ACTH, adrenocorticotropic hormone; AI, adrenal insufficiency; CD, Cushing disease; CS, Cushing syndrome; DOC, deoxycorticosterone; FC, fludrocortisone; Norm, normalization of cortisol secretion but no evidence of recurrence; Partial, normal basal cortisol but flat cosyntropin response; Rec., recurrence of hypercortisolism.

Imaging evidence of adrenal shrinkage has been reported in 2 cases of prolonged adrenal insufficiency following discontinuation of osilodrostat treatment [14, 15]. More recently, a retrospective ACTH-dependent Cushing syndrome series described adrenal volume reduction in all 10 evaluated patients treated with osilodrostat, 5 of whom developed adrenal insufficiency [18]. Among these 12 patients with reported adrenal shrinkage, only one had confirmed CD. In the retrospective series, adrenal volume reduction correlated with maximum osilodrostat dose [18], although its relationship to prolonged adrenal insufficiency remains uncertain.

The present case is distinguished not simply by the duration of adrenal insufficiency, but by the evolution toward prolonged pan-adrenal steroidogenic suppression. While glucocorticoid deficiency is an expected consequence of CYP11B1 inhibition, clinically significant mineralocorticoid deficiency would be less anticipated because of the compensatory mineralocorticoid activity of 11-deoxycorticosterone.

Initially, the patient demonstrated the expected biochemical profile of distal 11β-hydroxylase inhibition, with progressive accumulation of 11-deoxycortisol under rising ACTH concentrations. Once adrenal insufficiency developed, however, this pattern changed substantially. Despite marked ACTH elevation, 11-deoxycortisol was no longer increased, adrenal androgens became suppressed, and prolonged mineralocorticoid pathway impairment emerged. The transition from selective distal precursor accumulation to sustained suppression of glucocorticoid, androgen, and mineralocorticoid secretion suggests adrenal dysfunction extending beyond isolated CYP11B1 inhibition alone. Alternative causes of primary adrenal insufficiency in this patient appeared unlikely given the temporal relationship with prolonged osilodrostat exposure and the evolving biochemical profile.

The coexistence of pan-adrenal suppression and adrenal size reduction, notably in the context of chronically and markedly elevated ACTH concentrations, raises the possibility of structural adrenal remodeling after prolonged exposure. A conceptual parallel may exist with mitotane therapy, which is associated with progressive adrenal atrophy and prolonged adrenal insufficiency. Whether osilodrostat can induce a milder or mechanistically distinct form of adrenal remodeling remains unknown. Experimental studies have shown potent CYP11B1 and CYP11B2 inhibition together with partial CYP11A1 inhibition at higher concentrations [9], while in vitro adrenocortical models suggest broader steroidogenic alterations extending beyond distal precursor accumulation [9, 19]. Although long-term clinical data show a trend toward decreasing precursor levels with prolonged treatment, these remain substantially elevated even after 72 weeks [20], making the complete precursor suppression observed in our patient a qualitatively distinct finding. In contrast to the relative preservation of mineralocorticoid activity generally observed during osilodrostat treatment, our patient demonstrated persistent renin elevation requiring fludrocortisone replacement, suggesting more profound impairment of mineralocorticoid steroidogenesis. Current evidence nevertheless remains insufficient to establish a direct cytotoxic or adrenolytic effect in humans.

Although rare, prolonged adrenal suppression after discontinuation of osilodrostat warrants recognition. Recovery of adrenal function should not be assumed, and long-term follow-up may require assessment not only of glucocorticoid but also mineralocorticoid and adrenal androgen pathways, together with consideration of adrenal morphology in selected patients.

## Learning points

- Osilodrostat-induced steroidogenic suppression may involve all adrenal biosynthetic pathways, including glucocorticoid, androgen, and mineralocorticoid production.
- Bilateral adrenal size reduction may occur during osilodrostat treatment despite markedly elevated ACTH.
- Prolonged adrenal insufficiency after osilodrostat discontinuation is uncommon but can persist for years; recovery should not be assumed.
- Long-term follow-up should include assessment of all adrenal steroidogenic pathways and consideration of adrenal morphology in selected patients.

## Data Availability

Original data generated and analyzed during this study are included in this published article.

## Abbreviations

ACTH: adrenocorticotropic hormone
CD: Cushing disease
CS: Cushing syndrome
CT: computerized tomography
TSS: transsphenoidal surgery
UFC: urinary free cortisol

## References

[1] Fleseriu M, Auchus R, Bancos I, et al Consensus on diagnosis and management of Cushing's disease: a guideline update. Lancet Diabetes Endocrinol. 2021;9(12):847‐875. 34687601 10.1016/S2213-8587(21)00235-7 PMC8743006

[2] Natan DB, Serebro M, Ram Z, et al Prediction of remission and recurrence of Cushing's disease following transsphenoidal surgery (TSS): a single center, 20-year, retrospective series. Endocrine. 2025;90(1):256‐265. 40531432 10.1007/s12020-025-04304-w

[3] Mehta GU, Ding D, Patibandla MR, et al Stereotactic radiosurgery for Cushing disease: results of an international, multicenter study. J Clin Endocrinol Metab. 2017;102(11):4284‐4291. 28938462 10.1210/jc.2017-01385

[4] Guignat L, Bertherat J. Medical treatment of Cushing's syndrome. Endocrinol Metab (Seoul). 2025;40(1):26‐38. 39801039 10.3803/EnM.2024.501 PMC11898324

[5] Giustina A, Uygur MM, Frara S, et al Medical management pathways for Cushing's disease in pituitary tumors centers of excellence (PTCOEs). Pituitary. 2025;28(1):23. 39881009 10.1007/s11102-024-01485-x PMC11779774

[6] Pivonello R, Simeoli C, Di Paola N, Larocca A, Crescenzo EM, Colao A. Osilodrostat: a novel potent inhibitor of 11-Beta-Hydroxylase for the treatment of Cushing's syndrome. touchREV Endocrinol. 2024;20(1):43‐51. 38812665 10.17925/EE.2024.20.1.8 PMC11132648

[7] Sofer Y, Tordjman K, Osher E, Greenman Y, Stern N. Cushing's disease presenting as visual impairment. BMJ. 2014;348(feb12 1):g1217.

[8] Samson SL, Donegan D, Geer EB, et al Management of Osilodrostat therapy in patients with Cushing's syndrome: a modified Delphi consensus panel. J Endocr Soc. 2025;9(8):bvaf103. 40620482 10.1210/jendso/bvaf103 PMC12227144

[9] Valentín-Goyco J, Liu J, Peng HM, Oommen J, Auchus RJ. Selectivity of osilodrostat as an inhibitor of human steroidogenic cytochromes P450. J Steroid Biochem Mol Biol. 2023;231:106316. 37098354 10.1016/j.jsbmb.2023.106316 PMC10757358

[10] Bertagna X, Pivonello R, Fleseriu M, et al LCI699, a potent 11β-hydroxylase inhibitor, normalizes urinary cortisol in patients with Cushing's disease: results from a multicenter, proof-of-concept study. J Clin Endocrinol Metab. 2014;99(4):1375‐1383. 24423285 10.1210/jc.2013-2117

[11] Poirier J, Bonnet-Serrano F, Thomeret L, Bouys L, Bertherat J. Prolonged adrenocortical blockade following discontinuation of Osilodrostat. Eur J Endocrinol. 2023;188(6):K29‐K32. 37300549 10.1093/ejendo/lvad060

[12] Ferriere A, Salenave S, Puerto M, Young J, Tabarin A. Prolonged adrenal insufficiency following discontinuation of osilodrostat treatment for intense hypercortisolism. Eur J Endocrinol. 2024;190(1):L1‐L3. 38123490 10.1093/ejendo/lvad167

[13] Tejani S, Abramowitz J, Tritos NA, Hamidi O, Mirfakhraee S. Prolonged adrenal insufficiency after osilodrostat exposure with eventual recovery of adrenal function. JCEM Case Rep. 2024;2(6):luae088. 38832004 10.1210/jcemcr/luae088 PMC11146141

[14] Veloski C, Sturgeon A, Hallanger Johnson J. Prolonged adrenal insufficiency after failed cryoablation and osilodrostat for Cushing syndrome in nodular adrenal disease. JCEM Case Rep. 2025;3(6):luaf091. 40303510 10.1210/jcemcr/luaf091 PMC12037973

[15] Kaniuka-Jakubowska S, Kunc M, Maksymowicz M, et al A case of prolonged adrenal insufficiency following osilodrostat discontinuation with a review of the literature. Endocr J. 2026;73(1):87‐92. 41139506 10.1507/endocrj.EJ25-0150 PMC12819063

[16] Benderradji H, Walasiak F, Danais A, et al A rare case report of prolonged adrenal insufficiency following discontinuation of osilodrostat treatment for severe hypercortisolism due to ectopic ACTH syndrome. Presented at ECE-ESPE 2025. Endocrine Abstracts. 2025;110:EP654.

[17] Dzialach L, Respondek W, Siejka A, Witek P. Prolonged adrenal suppression after osilodrostat discontinuation in a patient with Cushing's disease with eventual hypercortisolism relapse: case report and literature review. Front Med (Lausanne). 2025;12:1629387. 41030260 10.3389/fmed.2025.1629387 PMC12477154

[18] Varlamov EV, Park BJ, Fleseriu M. Osilodrostat-associated adrenal gland shrinkage: a case series of patients with ACTH-dependent Cushing's syndrome. J Clin Endocrinol Metab. 2026;111(4):1084‐1090. 41054820 10.1210/clinem/dgaf552

[19] Creemers SG, Feelders RA, de Jong FH, et al Osilodrostat is a potential novel steroidogenesis inhibitor for the treatment of Cushing syndrome: an in vitro study. J Clin Endocrinol Metab. 2019;104(8):3437‐3449. 31127821 10.1210/jc.2019-00217

[20] Fleseriu M, Newell-Price J, Pivonello R, et al Long-term outcomes of osilodrostat in Cushing's disease: LINC 3 study extension. Eur J Endocrinol. 2022;187(4):531‐541. 35980235 10.1530/EJE-22-0317 PMC9513654

